# Post-traumatic stress disorder and adverse pregnancy outcomes: sexual orientation disparities in a prospective cohort study

**DOI:** 10.1186/s12884-026-08867-y

**Published:** 2026-03-02

**Authors:** Michelle W. Tam, Isa Berzansky, Payal Chakraborty, Colleen A. Reynolds, Kodiak R. S. Soled, Sarah McKetta, Cindy Veldhuis, Karestan Koenen, Brittany M. Charlton

**Affiliations:** 1 Department of Epidemiology, Harvard T.H. Chan School of Public Health, 677 Huntington Avenue, Boston, MA 02215 USA; 2 Dalla Lana School of Public Health, University of Toronto, Toronto, ON Canada; 3 Department of Population Medicine, Harvard Pilgrim Health Care Institute, Boston, USA; 4 Department of Social Medicine, University of North Carolina at Chapel Hill School of Medicine, Chapel Hill, NC USA; 5 Department of Public Health, Erasmus MC, University Medical Center Rotterdam, Rotterdam, South Holland Netherlands; 6 Department of Epidemiology, Mailman School of Public Health, Columbia University, New York City, NY USA; 8 Impact Institute, Northwestern University, Chicago, IL USA; 7 Department of Medical Social Sciences, Feinberg School of Medicine, Northwestern University, Chicago, IL USA; 9 Harvard Medical School, Boston, USA

**Keywords:** adverse pregnancy outcomes, gestational diabetes, gestational hypertension, preeclampsia, preterm birth, low birthweight, macrosomia, sexual orientation, United States

## Abstract

**Background:**

Post-traumatic stress disorder (PTSD) is a recognized risk factor for adverse pregnancy outcomes. Growing research indicates that sexual minority (SM) people face elevated risks for both PTSD symptoms/diagnosis and adverse pregnancy outcomes. However, no research has examined whether the association between sexual orientation and adverse pregnancy outcomes differs by PTSD symptoms/diagnosis.

**Methods:**

We used longitudinal data from the Nurses’ Health Study 3, a longitudinal cohort of nurses born on or after January 1, 1965, living in the US or Canada. We restricted analyses to those who reported their sexual orientation, completed the questionnaire with PTSD items, and had at least one pregnancy (*N* = 27,381 pregnancies from 10,090 participants). We examined whether the associations between sexual orientation and six adverse pregnancy outcomes) differed by PTSD symptoms/diagnosis prior to pregnancy across sexual orientation subgroups: completely heterosexual (reference), heterosexual with same-sex experience, mostly heterosexual, bisexual, and lesbian/gay. We used modified Poisson models to estimate risk ratios (RRs) for PTSD symptoms and diagnosis. Next, we examined whether sexual orientation and six adverse pregnancy outcomes (i.e., gestational diabetes, gestational hypertension, preeclampsia, preterm birth, low birthweight, macrosomia) varies by PTSD symptoms/diagnosis.

**Results:**

Compared to completely heterosexual participants, all SM subgroups had higher risks of PTSD symptoms prior to pregnancy (heterosexual with same-sex experience RR 1.23, 95% CI, 1.17–1.29; mostly heterosexual RR 1.37, 95% CI, 1.31–1.43; bisexual RR 1.58, 95% CI, 1.49– 1.67; lesbian/gay RR 1.46, 95% CI, 1.30 − 1.63) prior to pregnancy. SM participants (subgroups combined) had 2.6 times the risk of PTSD diagnosis (RR 2.64, 95% CI 1.38–5.05) compared to the completely heterosexual group. Sexual orientation disparities for low birthweight and preeclampsia were greater among those with, compared to without, PTSD symptoms; however, interaction tests of sexual orientation and PTSD symptoms were not significant. Pregnancies to SM participants (subgroups combined) with PTSD symptoms had a significantly higher risk of low birthweight than pregnancies to completely heterosexual participants; there was no significant difference among those without PTSD symptoms. Conversely, sexual orientation disparities in gestational hypertension were greater among pregnancies without PTSD symptoms, though the interaction was not significant.

**Conclusion:**

PTSD may lead to varied adverse pregnancy outcomes across sexual orientation groups. Screening for PTSD and using a trauma-informed approach to perinatal care is particularly critical for marginalized populations.

**Supplementary Information:**

The online version contains supplementary material available at 10.1186/s12884-026-08867-y.

## Introduction

Post-traumatic stress disorder (PTSD) affects 3%–19% of pregnant people across the US (1–3). PTSD contributes to poor obstetric and gynecological outcomes such as increased risk of unintended pregnancies (1–3), sexually transmitted infections (4, 5), chronic pelvic pain (6, 7), and conflicted feelings about pregnancy and sexuality (8). Compared to their heterosexual peers, sexual minority (SM) people experience disproportionate rates of trauma (8). Research reveals that SM people are disproportionately burdened by an increased risk for adverse childhood experiences, interpersonal violence, earlier trauma exposure, and PTSD (9–13). This population is more than twice as likely than their heterosexual peers to experience childhood maltreatment and subsequently develop PTSD (11). Given that SM people experience higher rates of trauma, the relationship between sexual orientation and adverse pregnancy outcomes may vary by presence of PTSD.

Specifically, PTSD prior to pregnancy is a known risk factor for adverse pregnancy outcomes such as preeclampsia, low birthweight, and preterm birth (14–17). In turn, both PTSD and adverse pregnancy outcomes contribute to postpartum mental health conditions, including postpartum depression and anxiety (17–21). For nearly five years, the American College of Obstetricians and Gynecologists has recommended all pregnant patients be universally screened for both current and past trauma (8). Therefore, we know trauma-informed care models need to be adapted in delivery of pregnancy care to meet the needs of all pregnant patient, including SM people who are disproportionately burdened with higher risk of trauma exposure (8). This prior work demonstrates a clear link between prior to pregnancy mental health (e.g., PTSD symptoms and diagnosis), delivery of prenatal care, and postpartum mental health.

A growing body of research shows that SM people experience an increased risks for various obstetric outcomes, such as unintended pregnancy (22, 23), adverse pregnancy outcomes (24, 25), and postpartum anxiety and depression (26, 27). Earlier studies led by members of our team have reported that SM people experience significantly higher risks of pregnancy complications such as gestational hypertension and preeclampsia (24), neonatal outcomes such as preterm birth and low birthweight (28), and adverse perinatal mental health such as pregnancy stress and depression (27). Furthermore, SM people experience barriers to perinatal care as a result of pervasive heteronormative assumptions regarding gametes, sexual activity, and family structures [29, 30]. Research consistently shows, discrimination and stigma are barriers for patients to access appropriate health and social services, leading to unmet healthcare needs for SM individuals (24, 37–41). Ongoing or repeated exposure to sexual orientation-based discrimination constitutes a form of minority stress that can threaten an individual’s sense of safety, be experienced as insidious trauma, and/or exacerbate existing PTSD and trauma symptoms (11, 31–33).

While sexual orientation disparities in adverse pregnancy outcomes are well documented, it remains unclear whether PTSD modifies this relationship (24, 25, 27, 28). Few studies have examined whether the relationship between sexual orientation and adverse pregnancy outcomes risks varies by prior to pregnancy mental health. Data on sexual orientation is seldom collected in health databases. As a result, there is limited visibility of obstetric outcomes among sexual minority people. Without a clearer understanding of the role of PTSD in adverse pregnancy outcomes, SM people are likely to continue having worse perinatal, neonatal, and postpartum mental health outcomes. This investigation is critical as mental health conditions prior to pregnancy and adverse pregnancy outcomes can contribute to poor postpartum outcomes (17–21).

To address this gap, we first assessed differences in PTSD symptoms and diagnoses prior to pregnancy across sexual orientation groups. We then examined whether the association between sexual orientation and adverse pregnancy outcomes varied by PTSD symptoms and diagnoses, testing for potential effect modification in these disparities. We used data from a large, prospective cohort of American and Canadian nurses to identify sexual orientation-related disparities regarding (1) PTSD symptoms and diagnosis prior to pregnancy; (2) adverse perinatal and neonatal outcomes among those with and without exposure to PTSD symptoms and diagnosis prior to pregnancy; and (3) whether associations of sexual-orientation and adverse pregnancy outcomes were modified by PTSD symptoms/diagnosis.

## Methods

### Data source

We analyzed data from the Nurses’ Health Study 3 (NHS3), a longitudinal cohort of nurses born on or after January 1, 1965, living in the US or Canada (*N* = 27,381 pregnancies from 10,090 participants). NHS3 is an ongoing cohort with enrollment that began in 2010. Upon enrollment, participants complete online questionnaires in sequence (starting with the 1st questionnaire) approximately every six months. This study was approved by the IRB of [blinded for peer review].

### Variables

Sexual orientation, including measures of identity, attraction, and sexual contact, was measured on the 5th, 10th, and 13th questionnaires using items adapted from the Minnesota Adolescent Health Survey (34). Using the most recent self-reports of sexual orientation, participants were asked to respond with the following options: completely heterosexual (attracted to persons of a different sex); mostly heterosexual; bisexual (attracted to men and women), mostly homosexual, and completely homosexual (gay/lesbian, attracted to persons of the same-sex), and not sure. Participants who reported “mostly homosexual” or “completely homosexual (lesbian/gay)”, were combined as the lesbian/gay group. Participants were categorized as heterosexual with same-sex experience if they reported “completely heterosexual” and either a prior sexual minority identity; having sexual contact with people who were the same-sex or another gender (e.g., gender fluid, non-binary); or being attracted to people of the same-sex or another gender (e.g., gender fluid, non-binary). We analyzed “mostly heterosexual” and “heterosexual with same‑sex experience” as separate groups because they may be exposed to different forms of minority stress (35). For example, heterosexual‑identified people with same‑sex experience may experience a mismatch between their sexual identity and behavior, which could incur minority stress that differs from that of mostly heterosexual individuals whose identity and behavior are more closely aligned. Additionally, prior research has shown that there are different health outcomes when comparing completely heterosexual women and heterosexual women with same-sex experiences (25, 36). We excluded pregnancies for participants if no data were available on sexual orientation.

We examined two PTSD-related outcomes. First, PTSD symptoms were reported on the 11th questionnaire (Table S1). Participants were asked to indicate PTSD symptoms, in reference to their worst traumatic event at the time of the questionnaire, according to the modified PTSD Checklist for DSM-5 (see Table S1; hereafter, PTSD symptoms). For that worst traumatic event, the traumatic events history was measured using a 16-item modified version of the Brief Trauma Questionnaire. Participants were asked to specify at what age their worst traumatic event occurred, which supported a temporality of PTSD symptoms for a worst traumatic event occurring prior to pregnancy. Second, lifetime PTSD diagnosis by a clinician, alongside other diagnoses, was asked on the 1st, 3rd, 5th, 7th, 9th, 11th, and 12th questionnaires. The year of diagnosis was also reported, which enabled a designation of a PTSD diagnosis prior to pregnancy.

Participants were asked detailed questions on lifetime pregnancies on the 1st and 13th questionnaires. The primary outcomes for this study were gestational diabetes, gestational hypertension, preeclampsia, preterm birth, low birthweight, and macrosomia. For preterm birth, low birthweight, and macrosomia, the analysis was restricted to live births. For gestational diabetes, gestational hypertension, and preeclampsia, the analysis was limited to pregnancies lasting 20 weeks or more, and subsequent pregnancies were excluded after the first occurrence of each respective outcome, as a history of gestational diabetes, gestational hypertension, and preeclampsia strongly predicts the recurrence of these conditions. Additionally, individuals with pre-existing chronic diabetes were excluded from the analysis of gestational diabetes, and those with pre-existing chronic hypertension were excluded from the analysis of gestational hypertension.

### Statistical analyses

Analysis occurred in two phases. First, we binarized PTSD symptoms and PTSD diagnosis into dichotomous variables. PTSD symptoms were dichotomized as: (1) no PTSD symptoms, including participants who reported no traumatic event or a traumatic event without symptoms; and (2) presence of PTSD symptoms. PTSD diagnosis is dichotomized as (1) PTSD diagnosis prior to pregnancy; and (2) no PTSD diagnosis prior to pregnancy. We used unadjusted modified Poisson models (McNutt et al., 2003) to estimate the risk ratio (RRs) of PTSD symptoms and PTSD diagnosis at the pregnancy-level. We used weighted generalized estimating equations (GEE) to account for multiple pregnancies from individuals, applying inverse cluster size weights to account for informative cluster sizes, treating each pregnancy as a separate observation, but weighting for repeated pregnancies within participants (37).

Consistent with our team’s established approach to mediators in sexual orientation-related analyses (38), we did not adjust for variables commonly adjusted for in reproductive health research (e.g., assisted reproductive technologies use, preconception health). Sexual orientation is a fundamental aspect of a person’s identity. Thus, adjusting for mediators may introduce bias and inhibit the mechanisms through which sexual orientation and adverse outcomes are linked (38–41). To account for potential confounding effects of age at pregnancy and year of pregnancy, we conducted sensitivity analyses.

In the second phase, for each pregnancy, we stratified by whether participants reported PTSD symptoms or PTSD diagnosis prior to pregnancy, and compared the risk of six adverse pregnancy outcomes (i.e., gestational diabetes, gestational hypertension, preeclampsia, preterm birth, low birthweight, macrosomia) across sexual orientation groups. We used modified Poisson models with weighted GEE, applying inverse cluster size weights, to estimate unadjusted RRs for adverse pregnancy outcomes. As a sensitivity analysis, we examined a higher PTSD symptoms threshold (≥ 3 PTSD symptoms) to align with a more conservative cutoff used in abbreviated PTSD screeners (42). To assess whether the association between PTSD variables and adverse pregnancy outcomes varied across sexual orientation groups, we conducted an interaction analysis using Wald tests.

## Results

### Analytic sample

Of the 51,071 NHS3 participants, 13,804 completed the 11th questionnaire where PTSD was measured; among these participants, 13,772 reported sexual orientation and 10,090 reported at least one pregnancy (*N* = 27,381 unique pregnancies). Among eligible participants, 4,231 were in the SM group, representing 41.5% of the unique pregnancies (Table 1).

**Table 1:** Individual- and pregnancy-level characteristics of participants in the Nurses’ Health Study 3

	Completely heterosexual^a^	Heterosexual with same-sex experience^b^	Mostly heterosexual	Bisexual	Lesbian/Gay
Individual-level					
	(58.07%, *n* = 5,859)	(17.58%, *n* = 1,774)	(18.76%, *n* = 1,893)	(4.21%, *n* = 425)	(1.38%, *n* = 139)
Race/Ethnicity, n (%)					
Asian, non-Hispanic	96 (1.64)	33 (1.86)	27 (1.43)	4 (0.94)	1 (0.72)
Black, non-Hispanic	103 (1.76)	40 (2.25)	23 (1.22)	6 (1.41)	2 (1.44)
Hispanic	189 (3.23)	73 (4.11)	79 (4.17)	16 (3.76)	5 (3.60)
Native American, non-Hispanic	15 (0.26)	7 (0.39)	3 (0.16)	1 (0.24)	0 (0.00)
Native Hawaiian/Pacific	8 (0.14)	3 (0.17)	2 (0.11)	1 (0.24)	0 (0.00)
Islander, non-Hispanic					
Middle Eastern/North African, non-Hispanic	3 (0.05)	2 (0.11)	4 (0.21)	0 (0.00)	0 (0.00)
White, non-Hispanic	5,328 (90.94)	1,565 (88.22)	1,688 (89.17)	365 (85.88)	129 (92.81)
Multiple/another, non-Hispanic	101 (1.72)	49 (2.76)	62 (3.28)	30 (7.06)	2 (1.44)
Missing (non-response)	16 (0.27)	2 (0.11)	5 (0.26)	2 (0.47)	0 (0.00)
Educational level, n (%)					
Post-secondary education	2,800 (47.79)	775 (43.69)	867 (45.80)	183 (43.06)	67 (48.20)
Graduate degree	3,059 (52.51)	999 (56.31)	1,026 (54.20)	241 (56.71)	72 (51.80)
Missing (non-response)	0 (0.00)	0 (0.00)	0 (0.00)	1 (0.24)	0 (0.00)
PTSD Symptoms^c^, n (%)	3,200 (60.73)	1,030 (71.48)	1,268 (76.94)	315 (86.54)	98 (81.67)
PTSD Diagnosis^d^, n (%)	146 (2.49)	63 (3.56)	111 (5.86)	41 (9.65)	16 (11.51)

	Completely heterosexual^a^	Heterosexual with same-sex experience^b^	Mostly heterosexual	Bisexual	Lesbian/Gay
Pregnancy-level					
	(58.52%, *n* = 16,023)	(17.86%, *n* = 4,891)	(18.47%, *n* = 5,058)	(4.08%, *n* = 1,117)	(1.07%, *n* = 292)
Age at pregnancy, mean years (SD), range (x-x)	29.6 (5.49), 12-50	29.3 (6.10), 12-49	29.9 (5.93), 13-49	29.4 (6.23) 13-49	29.3 (6.63) 15-42
12 – 17	302 (1.88)	153 (3.13)	137 (2.71)	45 (4.03)	13 (4.45)
18 – 24	2,445 (15.26)	914 (18.69)	821 (16.23)	211 (18.89)	56 (19.18)
25 – 34	10,295 (64.25)	2,854 (58.35)	2,963 (58.58)	616 (55.15)	159 (54.45)
35 – 39	2,417 (15.08)	743 (15.19)	928 (18.35)	200 (17.91)	49 (16.78)
>40	473 (2.95)	191 (3.91)	177 (3.50)	36 (3.22)	14 (4.79)
Missing	91 (0.57)	36 (0.74)	32 (0.63)	9 (0.81)	1 (0.34)
Gestational type, n (%)					
Singleton	11,703 (73.04)	3,268 (66.82)	3,254 (64.33)	679 (60.79)	183 (62.67)
Multiples	218 (1.36)	80 (1.64)	76 (1.50)	13 (1.16)	12 (4.11)
Non-live birth outcome^e^	4,026 (25.13)	1,513 (30.93)	1,695 (33.51)	417 (37.33)	97 (33.22)
Missing	76 (0.47)	30 (0.61)	33 (0.65)	8 (0.72)	0 (0.00)
Pregnancy outcome, n (%)					
Live birth	11,921 (74.40)	3,348 (68.45)	3,330 (65.84)	692 (61.95)	195 (66.78)
Stillbirth/miscarriage	2,933 (18.30)	937 (19.16)	1,042 (20.60)	238 (21.31)	53 (18.15)
Another outcome^f^	1,093 (6.82)	576 (11.78)	653 (12.91)	179 (16.03)	44 (15.07)
Missing	76 (0.47)	30 (0.61)	33 (0.65)	8 (0.72)	0 (0.00)
Adverse Pregnancy Outcome, n (%)					
Gestational diabetes^g^(18,870)	531 (4.61)	148 (4.53)	155 (4.80)	44 (6.72)	12 (6.35)
Gestational hypertensionh(18,280)	538 (4.78)	182 (5.89)	182 (5.87)	43 (6.63)	17 (9.60)
Pre-eclampsia^i^ (19,129)	479 (4.09)	201 (6.15)	162 (4.94)	35 (5.13)	10 (5.24)
Preterm birth (19,461)	1,004 (8.43)	298 (8.91)	249 (7.48)	65 (9.41)	19 (9.74)
Low birthweight (19,278)	510 (4.32)	174 (5.29)	158 (4.80)	36 (5.24)	14 (7.18)
Macrosomia (19,278)	2,791 (23.62)	714 (21.70)	760 (23.09)	155 (22.56)	65 (33.33)

Unadjusted comparisons across sexual orientation groups: PTSD diagnosis (Fisher’s exact test, p = 0.0005); PTSD symptom severity (χ² test, p = <0.0001); preeclampsia (χ² test, p = <0.0001); gestational hypertension (χ² test, p = 0.007); gestational diabetes (χ² test, p = 0.1083); macrosomia (χ² test, p = 0.0022); preterm birth (χ² test, p = 0.1878); low birthweight (χ² test, p = 0.04923)

^a^Completely heterosexual are participants who never had same-sex attractions, partners, or prior sexual minority identity

^b^Heterosexual with same-sex experience are participants who reported “completely heterosexual” and either a prior sexual minority identity; having sexual contact with people who were the same-sex or another gender (e.g. gender fluid, non-binary); or being attracted to people of the same-sex or another gender (e.g. gender fluid, non-binary)

^c^PTSD symptoms were assessed using modified PTSD Checklist for DSM-5 with at least 1 PTSD symptom, in reference to the participant’s self-identified worst traumatic event, including participants who completed the 11^th^questionnaire with a PTSD symptoms question

dPTSD diagnosis, person-level, lifetime, not prior to pregnancy, includes clinician-diagnosed PTSD within the past three years or any prior diagnosis more than three years ago

^e^Includes endorsement of a stillbirth, miscarriage, induced abortion, tubal, or ectopic pregnancy

^f^Includes endorsement of an induced abortion, tubal, or ectopic pregnancy

^g^Restricted to pregnancies at ≥20 weeks’ gestation, with non-missing diagnosis year and no history of chronic or gestational diabetes

^h^Restricted to pregnancies at ≥20 weeks’ gestation, with non-missing diagnosis year and no history of chronic hypertension or gestational hypertension

^i^Restricted to pregnancies at ≥20 weeks’ gestation, with non-missing diagnosis year and no history of preeclampsia

### Descriptive characteristics of the study sample

The prevalence of lifetime PTSD diagnosis (person-level) was higher among all SM subgroups— heterosexual with same-sex experience (3.6%), mostly heterosexual (5.9%), bisexual (9.7%), and lesbian/gay (11.5%)—compared to the completely heterosexual group (2.5%) (Table 1). All SM subgroups had a higher prevalence of PTSD symptoms (person-level, at least one symptom) with bisexual individuals having the highest prevalence (74.1%) followed by lesbian/gay (70.5%), mostly heterosexual (67.0%), heterosexual with same-sex experience (58.1%), compared to the completely heterosexual group (54.6%). Compared to the completely heterosexual group, pregnancies of all SM subgroups had higher prevalences of gestational hypertension, preeclampsia, and low birthweight. Pregnancies among lesbian/gay individuals had the highest prevalence of gestational hypertension (9.6%), preterm birth (9.7%), low birthweight (7.2%), and macrosomia (33.3%).

In the analytic sample, we observed unadjusted differences in PTSD symptom and PTSD diagnosis severity across sexual orientation groups (symptoms: *p* < 0.0001; diagnosis: *p* = 0.0005), as well as differences in adverse pregnancy outcomes (p-values for individual outcomes ranging from 0.188 to < 0.0001; see Table 1). These comparisons are descriptive and unadjusted; adjusted associations are presented in subsequent models.

### Differences in trauma and PTSD by sexual orientation

Compared to completely heterosexual participants, SM participants (all subgroups combined) had a higher risk of PTSD symptoms (risk ratio [RR] 1.34, 95% confidence interval [CI], 1.29–1.39) and diagnosis (RR 2.64, 95% CI, 1.38–5.05) (Table 2). When these outcomes were stratified across sexual orientation groups, RRs were statistically significant for PTSD symptoms across all SM subgroups (heterosexual with same-sex experience RR 1.23, 95% CI, 1.17–1.29; mostly heterosexual RR 1.37, 95% CI, 1.31–1.43; bisexual RR 1.58, 95% CI, 1.49–1.67; lesbian/gay RR 1.46, 95% CI, 1.30–1.63). For PTSD diagnosis, SM participants (all subgroups combined) were 2.6 times more at risk compared to the completely heterosexual group, driven by mostly heterosexual (RR 3.19, 95% CI 1.52–6.68) and bisexual (RR 8.35, 95% CI 3.52–19.78) individuals. Stratified analysis for the subgroup of lesbian individuals with PTSD diagnosis prior to pregnancy was not performed due to absence of eligible participants.

**Table 2:** Risk ratios of PTSD symptoms, and PTSD diagnosis prior to pregnancy across sexual orientation groups

	Completely heterosexual (reference)	Sexual minority	Heterosexual with same-sex experience	Mostly heterosexual	Bisexual	Lesbian
	Pregnancy-level Risk ratio (95% confidence interval)					
PTSD Symptoms^a^	(*n* = 10,783)	(*n* = 7,016)	(*n* = 2,864)	(*n* = 3,268)	(*n* = 699)	(*n* = 185)
	1.00 (ref)	1.34 (1.29, 1.39)	1.23 (1.17, 1.29)	1.37 (1.31, 1.43)	1.58 (1.49, 1.67)	1.46 (1.30, 1.63)
	Pregnancy-level Risk ratio (95% confidence interval)					
PTSD Diagnosis^b^	(*n* = 15,642)	(*n* = 10,759)	(*n* = 4,705)	(*n* = 4,773)	(*n* = 1,021)	(*n* = 260)
	1.00 (ref)	2.64 (1.38, 5.05)	0.95 (0.31, 2.89)	3.19 (1.52, 6.68)	8.35 (3.52, 19.78)	--^c^

^a^PTSD symptoms prior to pregnancy were assessed using modified PTSD Checklist for DSM-5, in reference to the participant’s self-identified worst traumatic event

^b^PTSD diagnosis prior to pregnancy includes clinician-diagnosed PTSD within the past three years or any prior diagnosis more than three years ago

^c^ No lesbian participants met eligibility criteria for the subgroup with PTSD diagnosis prior to pregnancy in the risk ratio models; therefore, estimates for this subgroup could not be calculated. Lesbian participants with PTSD diagnosis are, however, included in the descriptive prevalence estimates in Table 1 (*n* = 16)

### Adverse pregnancy outcomes by sexual orientation by strata of PTSD symptoms

**Fig. 1:**
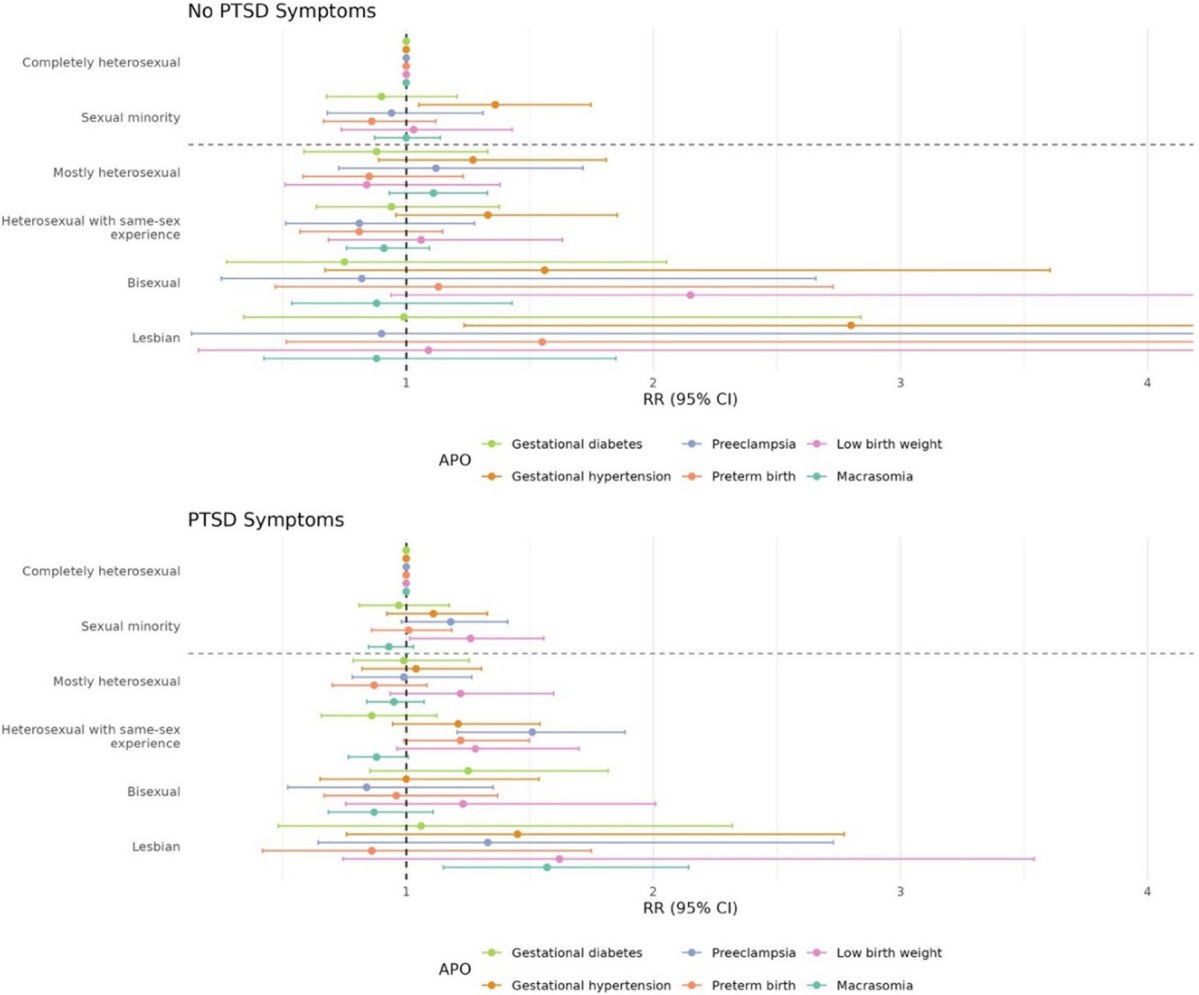
Forest plot for risk ratios of adverse pregnancy outcomes by PTSD symptoms^a^ prior to pregnancy across sexual orientation groups^a, b,c, d^. Full risk ratio and sample sizes are available in Table 3 ^a^PTSD symptoms prior to pregnancy were assessed using modified PTSD Checklist for DSM-5, in reference to the participant’s self-identified worst traumatic event. No PTSD symptoms includes participants who reported no traumatic event and participants who reported a traumatic event with no PTSD symptoms. ^b^Completely heterosexual are participants who never had same-sex attractions, partners, or prior sexual minority identity; sexual minority includes the following subgroups: heterosexual with same-sex experience, mostly heterosexual, bisexual, and lesbian/gay; heterosexual with same-sex experience are participants who reported “completely heterosexual” and either a prior sexual minority identity; having sexual contact with people who were the same-sex or another gender (e.g. gender fluid, non-binary); or being attracted to people of the same-sex or another gender (e.g. gender fluid, non-binary). ^c^Gestational diabetes is restricted to pregnancies at ≥20 weeks’ gestation, with diagnosis year and no history of chronic or gestational diabetes; gestational hypertension is restricted to pregnancies at ≥20 weeks’ gestation, with diagnosis year and no history of chronic hypertension or gestational hypertension; preeclampsia is restricted to pregnancies at ≥20 weeks’ gestation, with diagnosis year and no history of preeclampsia. ^d^Wald tests for group comparisons: χ2 = 0.85, df = 4, *p* = 0.93 for gestational diabetes; χ2= 2.6, df =4, *p* = 0.63 for gestational hypertension; χ2 = 7.9, df = 4, *p* = 0.095 for preeclampsia; χ2 = 5.3, df =4, *p* = 0.26 for preterm birth; χ2 = 5.1, df = 4, *p* = 0.28 for low birthweight; χ2 = 4.5, df = 4, *p* = 0.34 for macrosomia

Sexual orientation disparities in preeclampsia and low birthweight were generally greater among those with, compared to those without, PTSD symptoms; the exceptions are for mostly heterosexual individuals for preeclampsia and bisexual individuals for low birth weight (Table 3; Fig. 1). Specifically, among those who reported PTSD symptoms prior to pregnancy, pregnancies to SM participants compared to heterosexual peers had a higher risk of low birthweight (RR 1.26, 95% CI, 1.01–1.56); no significant differences were observed among those without PTSD symptoms (RR 1.03, 95% CI, 0.74–1.43). Furthermore, compared to completely heterosexual participants, SM participants (subgroups combined) who reported PTSD symptoms, the RR for preeclampsia (RR 1.18, 95% CI 0.98–1.41) was elevated, although not statistically significant, while we saw no differences among those with no symptoms (RR 0.94, 95% CI 0.68–1.31). Notably, the higher risk of preeclampsia among those who reported PTSD symptoms was primarily driven by heterosexual participants with same-sex experience (RR 1.51, 95% CI 1.21–1.88). Additionally, pregnancies of lesbian/gay individuals with PTSD symptoms were associated with a statistically significant RR of 1.57 (95% CI, 1.15–2.14) for macrosomia, while no significant differences were observed among those without PTSD symptoms (RR 0.88, 95% C 0.42–1.85). Formal interaction tests were not statistically significant for preeclampsia (χ^2^(4) = 7.9, *p* = 0.095), low birthweight χ^2^(4) = 5.1, *p* = 0.28) or macrosomia (χ^2^(4) = 4.5, *p* = 0.34).

**Table 3:** Risk ratios of adverse pregnancy outcomes by PTSD symptoms^a^ across sexual orientation groups

	Completely heterosexual^b^ (reference) (*n* = 10,783)	Sexual minority^c^ (*n* = 7,016)	Heterosexual with same-sex experience^d^ (*n* = 2,864)	Mostly heterosexual (*n* = 3,268)	Bisexual (*n* = 699)	Lesbian (*n* = 185)
No PTSD Symptoms Risk ratio (95% confidence interval)						
Gestational diabetes^e^ (*n* = 5,548)	1.00 (ref)	0.90 (0.68, 1.21)	0.94 (0.64, 1.38)	0.88 (0.59, 1.33)	0.75 (0.27, 2.05)	0.99 (0.34, 2.84)
Gestational hypertension^f^ (*n* = 5,413)	1.00 (ref)	1.36 (1.06, 1.75)	1.33 (0.96, 1.85)	1.27 (0.89, 1.81)	1.56 (0.67, 3.60)	2.80 (1.23, 6.35)
Preeclampsia^g^ (*n* = 5,650)	1.00 (ref)	0.94 (0.68, 1.31)	0.81 (0.51, 1.28)	1.12 (0.73, 1.72)	0.82 (0.25, 2.66)	0.90 (0.13, 6.12)
Preterm birth (*n* = 5,738)	1.00 (ref)	0.86 (0.67, 1.12)	0.81 (0.57, 1.15)	0.85 (0.58, 1.23)	1.13 (0.47, 2.73)	1.55 (0.51, 4.67)
Low birthweight (*n* = 5,718)	1.00 (ref)	1.03 (0.74, 1.43)	1.06 (0.68, 1.63)	0.84 (0.51, 1.38)	2.15 (0.94, 4.92)	1.09 (0.16, 7.42)
Macrosomia (*n* = 5,718)	1.00 (ref)	1.00 (0.87, 1.14)	0.91 (0.76, 1.09)	1.11 (0.93, 1.33)	0.88 (0.54, 1.43)	0.88 (0.42, 1.85)
PTSD Symptoms Risk ratio (95% confidence interval)						
Gestational diabetes^e^ (*n* = 8,388)	1.00 (ref)	0.97 (0.81, 1.17)	0.86 (0.66, 1.12)	0.99 (0.79, 1.25)	1.25 (0.85, 1.82)	1.06 (0.48, 2.32)
Gestational hypertension^f^ (*n* = 8,072)	1.00 (ref)	1.11 (0.92, 1.33)	1.21 (0.95, 1.54)	1.04 (0.82, 1.30)	1.00 (0.65, 1.54)	1.45 (0.76, 2.77)
Preeclampsia^g^ (*n* = 8,517)	1.00 (ref)	1.18 (0.98, 1.41)	1.51 (1.21, 1.88)	0.99 (0.78, 1.26)	0.84 (0.52, 1.35)	1.33 (0.64, 2.73)
Preterm birth (*n* = 8,699)	1.00 (ref)	1.01 (0.86, 1.18)	1.22 (0.99, 1.50)	0.87 (0.70, 1.08)	0.96 (0.67, 1.37)	0.86 (0.42, 1.75)
Low birthweight (*n* = 8,654)	1.00 (ref)	1.26 (1.01, 1.56)	1.28 (0.96, 1.70)	1.22 (0.93, 1.60)	1.23 (0.76, 2.01)	1.62 (0.74, 3.54)
Macrosomia (*n* = 8,654)	1.00 (ref)	0.93 (0.85, 1.03)	0.88 (0.77, 1.01)	0.95 (0.84, 1.07)	0.87 (0.69, 1.11)	1.57 (1.15, 2.14)

^a^PTSD symptoms prior to pregnancy were assessed using modified PTSD Checklist for DSM-5, in reference to the participant’s self-identified worst traumatic event. No PTSD symptoms includes participants who reported no traumatic event and participants who reported a traumatic event with no PTSD symptoms

^b^Completely heterosexual are participants who never had same-sex attractions, partners, or prior sexual minority identity

^c^Sexual minority includes the following subgroups: heterosexual with same-sex experience, mostly heterosexual, bisexual, and lesbian/gay

^d^Heterosexual with same-sex experience are participants who reported “completely heterosexual” and either a prior sexual minority identity; having sexual contact with people who were the same-sex or another gender (e.g. gender fluid, non-binary); or being attracted to people of the same-sex or another gender (e.g. gender fluid, non-binary)

^e^Restricted to pregnancies at ≥20 weeks’ gestation, with diagnosis year and no history of chronic or gestational diabetes

^f^Restricted to pregnancies at ≥20 weeks’ gestation, with diagnosis year and no history of chronic hypertension or gestational hypertension

^g^Restricted to pregnancies at ≥20 weeks’ gestation, with diagnosis year and no history of preeclampsia

Wald tests for group comparisons:

Gestational diabetes: χ^2^= 0.85, df = 4, p = 0.93

Gestational hypertension: χ^2^= 2.6, df =4, p = 0.63

Preeclampsia: χ^2^= 7.9, df = 4, p = 0.095

Preterm birth: χ^2^ = 5.3, df =4, p = 0.26

Low birthweight: χ^2^ = 5.1, df = 4, p = 0.28

Macrosomia: χ^2^ = 4.5, df = 4, p = 0.34

In contrast, sexual orientation disparities in gestational hypertension were greater among pregnancies without PTSD symptoms compared to those with symptoms; though, of note, the formal interaction tests were not statistically significant (χ^2^(4) = 2.6, *p* = 0.63). Particularly, among those without PTSD symptoms, SM participants had a higher risk than heterosexual peers for gestational hypertension (RR 1.36, 95% CI, 1.06–1.75). RR magnitudes appeared elevated for all SM subgroups, but were only statistically significant for lesbian/gay participants. RRs were lower for those with PTSD symptoms.

Results from sensitivity analyses adjusted for age and year of pregnancy were similar to those from the main analysis (Table S2). Additionally, we conducted a secondary analysis using a higher threshold of ≥ 3, rather than ≥ 1, PTSD symptoms (Table S3). Results remained suggestive of heterogeneous effects across the two groups, though the patterns were less pronounced. Overall, Wald tests for interaction were not statistically significant (all p‑values > 0.05), indicating little statistical evidence that these associations varied by PTSD status.

### Adverse pregnancy outcomes by sexual orientation by strata of PTSD diagnosis

For PTSD diagnosis, although the analyses on pregnancy outcomes by sexual orientation were underpowered to detect differences, we present the findings in the Supplementary Materials (Table S4) to aid interpretation and support future investigation. Results indicate heterogeneous associations, with risk ratios varying considerably by PTSD diagnosis status. The observed associations for those with PTSD diagnosis were not statistically significant and had wide confidence intervals. There were no participants in the lesbian group with PTSD diagnosis and APO to conduct analysis, which constrains interpretation of the results and highlights sample size limitations (Table S4).

## Discussion

In the present study, we tested differences in PTSD symptoms and diagnosis prior to pregnancy across sexual orientation groups, and whether the association between sexual orientation and adverse pregnancy outcomes varies by PTSD symptoms or diagnoses within a large national cohort. Findings indicate that all SM subgroups (with outcomes) are significantly more likely to experience PTSD symptoms and be diagnosed with PTSD prior to pregnancy compared to their completely heterosexual counterparts; the one exception was among heterosexual people with same-sex experience and PTSD diagnosis. Notably, bisexual individuals are more than eight times as likely, and mostly heterosexual individuals are over three times as likely to have a PTSD diagnosis prior to pregnancy when compared to completely heterosexual individuals. For differences in adverse pregnancy outcomes, we found that pregnancies among SM individuals (combined groups) with PTSD symptoms prior to pregnancy are at a significantly higher risk of experiencing low birthweight. At the same time, there were no significant differences among those without PTSD symptoms. Pregnancies among most SM subgroups with PTSD symptoms prior to pregnancy were associated with elevated risks of gestational hypertension and preeclampsia; however, not all associations achieved statistical significance.

Our findings on PTSD diagnoses prior to pregnancy and adverse pregnancy outcomes underscore the importance of examining SM subgroups with greater specificity, even when observed differences in PTSD risk are modest. Although the confidence intervals are wide and the results should be interpreted with caution, the direction of the associations suggest that SM people with PTSD diagnoses may be at an increased risk for gestational diabetes, gestational hypertension, preeclampsia, and low birthweight. The broad confidence intervals underscore the uncertainty of the findings and the need for replication in adequately powered studies. Few existing studies have examined PTSD across SM subgroups, as there is a lack of routine sexual orientation data collection in health databases (43). Some evidence suggests that bisexual women have higher rates of probable diagnosis of PTSD compared to lesbian women (31). Additionally, for pregnancy outcomes, there is evidence of heterogeneity in low birthweight outcomes across subgroups of SM people (24, 28). Together, these patterns in PTSD diagnosis and adverse pregnancy outcomes—in prior work and the present study—highlight potential heterogeneity within SM subgroups and point to important directions for future research.

In our interaction analysis, we found little statistical evidence that sexual orientation and adverse pregnancy outcomes associations differed by PTSD status. Rather than diminishing the factors of PTSD, the finding suggest that PTSD may contribute to adverse pregnancy outcomes in broadly similar ways across sexual orientation groups. While PTSD may not moderate sexual orientation disparities in adverse pregnancy outcomes (differential impact) the high burden of PTSD among sexual minority people may still mediate these disparities.

Building on this, our findings reinforce previous findings on the increased burden on SM people for trauma exposure and risk of PTSD (11, 44). In comparison to their heterosexual counterparts, SM people disproportionately experience violence across their lifetimes, particularly interpersonal violence, sexual violence, and adverse childhood experiences (9–11, 13, 44–46). SM people experience minority stress, a form of chronic stress that leads to health disparities arising from the stigma, discrimination, and prejudice they encounter due to their sexual identity (47–49). SM people face systemic discrimination and violence in various environments, such as schools (50–52), healthcare facilities (53–55), workplaces (56–58), and public spaces (59, 60). Exposure to minority stress has been associated with changes in biological outcomes (61, 62), including cortisol (63–65), immune function (66, 67), and cardiovascular function (61, 66, 68–70).

In our study, participants self-reported having a PTSD diagnosis from a clinician. Even without the confirmation via a medical record, the sample size led to limited statistical power. Nonetheless, it is important to consider that PTSD is frequently underdiagnosed and underreported due to underscreening, inability to access mental healthcare, avoidance behaviors, dissociation, lack of awareness, fear of judgement, and/or memory issues (71, 72). For SM people, diagnosis of PTSD may be underreported, given that receiving a diagnosis requires having received mental health care after a trauma, and rates of trauma treatment vary by sexual identity (between 18 and 50%) and other unmeasured factors that support or inhibit obtaining mental healthcare (73). Previous research suggests that among SM people, there are sizable differences in use of mental healthcare by race/ethnicity and sexual identity (73). Further, despite the overall high use of mental healthcare among SM people, there are large unmet needs for mental health care.

This is the first study, to our knowledge, to look at adverse pregnancy outcomes across sexual orientation subgroups with exposure to PTSD symptoms and diagnosis prior to pregnancy. PTSD as a chronic stress response, leads to a continuous release of stress hormones like cortisol and adrenaline. This prolonged state of heightened stress and cortisol levels can have detrimental effects on health, including adverse pregnancy outcomes (16, 74–80). Most research on pre-pregnancy mental health and adverse pregnancy outcomes has primarily focused on depression, anxiety, mood, stress, and life events (79, 81–85). A review on preconception and prenatal stress revealed that preconception stress, along with exposure to severe life events, was linked to preterm birth and being small for gestational age (79, 82, 83, 85). This suggests the need for research to examine preconception mental health, as a sole focus on pregnancy stress may overlook critical pathways to adverse pregnancy outcomes (79). Our findings contribute to the limited research on pre-pregnancy PTSD symptomatology, confirming an increased risk of adverse pregnancy outcomes among SM groups with a diagnosis of pre-pregnancy PTSD and related symptoms. Furthermore, this study provides nuanced insights and stratifies risk by sexual orientation, highlighting the connection between chronic stressors (e.g., minority stressors) and adverse pregnancy outcomes.

There are many mechanisms that could explain our findings. For example, we found that among those with PTSD symptoms, pregnancies to SM people had a significantly higher risk of low birthweight compared with completely heterosexual people. Chronic stress is associated with dysregulation of the hypothalamic-pituitary-adrenal (HPA) axis and elevated cortisol levels (86), which in turn are associated with increased risk of low birthweight (87–89). Additionally, guided by the SM stress theory, SM people experience heightened stress compared to completely heterosexual people, stemming from stigma and discrimination (47, 49). Similarly, our findings were suggestive of disparities for gestational hypertension and preeclampsia—which has also been found to be related to the HPA axis (87–89)—though statistical power was limited. Furthermore, chronic stress operates through differences in health behaviors. SM people have higher rates of smoking, alcohol and substance use, and are more likely to delay seeking healthcare compared to their heterosexual peers (90, 91). In addition to higher rates of trauma exposure, they also experience a greater prevalence of depression and anxiety (91). These factors may also contribute to the disparities we found.

Unequal access to perinatal care remains a key concern for SM individuals, often driven by discrimination and stigma within healthcare systems, including heteronormativity and homophobia. Existing research on pre-conception and perinatal care indicates that SM people experience poor quality of care, discrimination, and traumatization (92, 93). In terms of poor quality of care, SM people report healthcare staff having limited knowledge of parenthood pathways for SM people (92, 94–100), absence of transparent conversations and informed consent (92, 97, 101–103), as well as exclusion from decision making (92, 101, 104–106). SM people experience a myriad of discrimination in fertility and perinatal care through heterosexist assumptions about their relationships and sexuality (92, 96, 100, 104, 107–109), exclusion of non-gestational parents (92, 96, 99, 104, 109–113), and refusal of services and care (92, 96, 107, 108, 113, 114). These negative and stigmatizing perinatal healthcare experiences may lead to or exacerbate postpartum mental health issues and negatively impact willingness to seek postpartum care.

Given association of PTSD symptoms and adverse pregnancy outcomes among SM individuals, along with accompanying disparities across sexual orientation groups, our findings underscore the need for a multi-pronged approach. One such approach could be the adoption of LGBTQ+ competent, trauma-informed care in conception, perinatal, and postpartum healthcare (8, 115, 116). Often unaddressed, screening for trauma and PTSD during the perinatal and postpartum periods can enable early intervention and risk mitigation of adverse pregnancy outcomes and postpartum PTSD (8, 15, 116, 117). Additionally, experiences in pregnancy and perinatal care can exacerbate existing PTSD or trigger new symptom onset (116). ACOG recommends that perinatal service providers be familiar with trauma-informed care models (8). These providers may offer trauma-specific services or referrals related to sexual abuse or assault. However, a trauma-informed model of care is distinct in that it adopts a universal and strengths-based approach. This model focuses on understanding the effects of trauma and aims to ensure that organizations, services, and providers foster a sense of safety for both patients and staff (118). An LGBTQ+ competent trauma-informed care model should recognize that various forms of discrimination, oppression, and prejudice experienced by LGBTQ+ communities can uniquely impact health, access to healthcare services, and the overall experience of those services (115). It also involves reflecting on and unlearning of heterosexist, cissexist assumptions and biases that are harmful to LGBTQ+ communities (115).

### Limitations

This study is the first to our knowledge to investigate whether sexual orientation-related disparities in adverse pregnancy outcomes differs by PTSD symptoms/diagnosis. However, there are several limitations. First, the sample was relatively homogenous in terms of race/ethnicity, educational level, and likely homogeneous in terms of health literacy as nurses compared to the general population. The NHS3 cohort predominantly consists of non-Hispanic White (> 90%) nurses with at least a post-secondary degree, which limits generalizability. Participants may have greater knowledge of perinatal health and mental health resources compared to the general population. Consequently, this study may underestimate adverse pregnancy disparities.

The lack of routine sexual orientation data collection in health databases limits the identification of obstetric and gynecological outcomes among SM people. Although the NHS3 is a large national cohort, sample size and statistical power remain limitations. This underscores the need to not only preserve existing cohorts with sexual orientation data, but also the need to incorporate sexual orientation measures in large cohorts and administrative datasets to better analyze mechanisms and modifiers of sexual orientation disparities in adverse pregnancy outcomes.

We examined PTSD symptoms and diagnosis that relied on participant recall of events, symptoms, and diagnosis. This may result in participants misremembering or misreporting. We were unable to assess PTSD diagnosis prior to pregnancy using the severity and cut-off scores of the modified PTSD Checklist for DSM-5, as symptom severity was measured based on the past month relative to when the questionnaire was completed, rather than during the pregnancy period. As a result, we utilized participants’ self-reported PTSD diagnosis by a clinician. Nonetheless, prior research has shown that subjective measures (self-report) of trauma are more strongly associated with the PTSD risk, when compared to objective measures (119, 120). A recent systematic review has demonstrated that retrospective measures (i.e., self-report, memory recall) of childhood maltreatment are more strongly associated with mental health diagnoses or symptoms than prospective measures (i.e., parents, court records, child protection records).

Another limitation on this study is that the temporality of PTSD symptoms and diagnosis was assessed as lifetime, rather than closest to pregnancy. We dichotomized PTSD symptoms prior to pregnancy, which does not account for symptom severity or treat symptoms as a continuous variable. Additionally, to establish PTSD symptoms temporality prior to pregnancy, we utilized reporting of symptoms in reference to the worst traumatic event at the time of the questionnaire and age at the worst traumatic event. This may have missed other instances of traumatic events and resultant PTSD symptoms. For instance, designation of the worst traumatic event may differ throughout one’s lifespan, such that a worst traumatic event prior to pregnancy may have occurred, but an even worse traumatic event happened post-pregnancy. Depending on timing of questionnaire completion, it may be possible that only the post-pregnancy worst traumatic event was captured and not the worst traumatic event prior to pregnancy. Thus, our reporting of PTSD symptoms may be underreported as it may be missing traumatic events not reported as worst at time of pregnancy (71–73).

## Conclusion

Using individual- and pregnancy-level data from a large national cohort, we found that SM people have a higher risk than their heterosexual peers of having PTSD symptoms and diagnosis prior to pregnancy. Among participants with PTSD symptoms or diagnosis prior to pregnancy, we also observed a higher risk of among SM people than their heterosexual peers of preeclampsia and low birthweight. Our findings highlight the importance of screening for trauma and PTSD, as well as incorporating trauma-informed approaches to perinatal care. Additionally, further research is essential in examining the various types of trauma—such as adverse childhood experiences, interpersonal violence, sexual violence, and other forms—that SM individuals are at a high risk of encountering throughout their lives and how these experiences affect perinatal and postpartum health. Future research should focus on the mechanisms that contribute to sexual orientation-related inequities, such as discrimination and minority stress, in the context of trauma, PTSD, and adverse pregnancy outcomes.

## Supplementary Information

Supplementary Material 1.

## Data Availability

The data that support the findings of this study are available on request from the Channing Division of Network Medicine at Brigham and Women’s Hospital and Harvard Medical School. The data are not publicly available due to privacy or ethical restrictions.
