# Supplementary material for: Post-traumatic stress disorder and adverse pregnancy outcomes: sexual orientation disparities in a prospective cohort study
Source: BMC Pregnancy Childbirth. 2026 Mar 2;26:378. doi: 10.1186/s12884-026-08867-y (PMC13059231; doi:10.1186/s12884-026-08867-y)
Supplement: Supplementary file 1 — Supplementary Material 1. [file 12884_2026_8867_MOESM1_ESM.docx]

Table S1. Survey questions and responses collected for sexual orientation, PTSD, and adverse pregnancy outcomes variables in the Nurses’ Health Study 3.

| **Variable** | **Question** | **Response Options** | **Questionnaires Collected** |
| --- | --- | --- | --- |
| Sexual Identity | Which one of the following best describes your feelings? | 1. Completely heterosexual (attracted to persons of the opposite sex) 2. Mostly heterosexual 3. Bisexual (equally attracted to men and women) 4. Mostly homosexual 5. Completely homosexual (gay/lesbian, attracted to persons of the same sex) 6. Not sure | 5.1, 5.2 10.1, 10.2, 13 |
|  | During your life, have you EVER identified yourself as mostly heterosexual, bisexual, or lesbian or gay? | 1. Yes 2. No | 10.1 |
| Sexual Partner(s) | During your life, the person(s) with whom you have had sexual contact  (however you define it) is (are): | 1. I have not had sexual contact with anyone 2. Female(s) 3. Male(s) 4. Female(s) and male(s) | 5.1 |
|  | During your lifetime, have you EVER had sexual contact with a female? | 1. Yes 2. No | 10.1 |
|  | During your lifetime, how many different women have you had sexual contact (however you define it)? | 1. 0 2. 1 3. 2 4. 3-5 5. 6-10 6. 11-14 7. 15-24 8. 25-34 9. 35+ | 13 |
|  | During your lifetime, how many different people with another gender (such as gender fluid, non-binary) have you had sexual contact (however you define it)? | 1. 0 2. 1 3. 2 4. 3-5 5. 6-10 6. 11-14 7. 15-24 8. 25-34 9. 35+ | 13 |
| Sexual Attraction | There are many ways people are sexually attracted to other people. Which best describes your current feelings? | 1. Only attracted to men 2. Mostly attracted to men 3. Equally attracted to men and women 4. Mostly attracted to women 5. Only attracted to women 6. Not attracted to other people 7. Not sure | 5.2, 10.2, 13 |
|  | During your lifetime, have you EVER been sexually attracted to females? | 1. Yes 2. No | 10.1 |
|  | During your lifetime, have you EVER been sexually attracted to women? | 1. Yes 2. No | 13 |
|  | During your lifetime, have you EVER been sexually attracted to people with another gender (such as gender fluid, non-binary)? | 1. Yes 2. No | 13 |
| PTSD Diagnosis^a^ | Please select any clinician diagnoses or procedures you have had in the past 3 years: | PTSD [yes/no] | 1.5, 3.3, 5.2, 7.1, 9.3, 11.2, 12 |
|  | What year were you first diagnosed with PTSD? | Fill-in-the blank | 1.5, 3.3, 3.4, 5.2, 7.2, 9.3, 11.2, 12 |
| PTSD Symptoms | Keeping the worst event in mind, indicate if you have EVER been bothered by the problems below: | 1. Repeated, disturbing, and unwanted memories of the stressful experience? 2. Repeated, disturbing dreams of the stressful experience? 3. Suddenly feeling or acting as if the stressful experience were actually happening again (as if you were actually back there reliving it)? 4. Feeling very upset when something reminded you of the stressful experience? 5. Having strong physical reactions when something reminded you of the stressful experience (for example, heart pounding, trouble breathing, sweating)? 6. Avoiding memories, thoughts, or feelings related to the stressful experience? 7. Avoiding external reminders of the stressful experience (for example, people, places, conversations, activities, objects, or situations)? 8. Trouble remembering important parts of the stressful experience? 9. Having strong negative beliefs about yourself, other people, or the world (for example, having thoughts such as: I am bad, there is something seriously wrong with me, no one can be trusted, the world is completely dangerous)? 10. Blaming yourself or someone else for the stressful experience or what happened after it? 11. Having strong negative feelings such as fear, horror, anger, guilt, or shame? 12. Loss of interest in activities that you used to enjoy? 13. Feeling distant or cut off from other people? 14. Trouble experiencing positive feelings (for example, being unable to feel happiness or have loving feelings for people close to you)? 15. Irritable behavior, angry outbursts, or acting aggressively? 16. Taking too many risks or doing things that could cause you harm? 17. Being “super alert” or watchful or on guard? 18. Feeling jumpy or easily startled? 19. Having difficulty concentrating? 20. Trouble falling or staying asleep? 21. Felt that there was no point in planning for the future? 22. None of the above. | 11.1 |
|  | How old were you when your worst experience occurred? | Fill-in-the-blank | 11.1 |
| Gestational Diabetes, Gestation Hypertension, Prececlampsia | Did you have any of these complications related to pregnancy or lactation during this pregnancy? | 1. None 2. Gestational diabetes 3. Pregnancy-related high blood pressure 4. Preeclampsia/toxemia 5. Hemorrhage (heavy bleeding) 6. Mastitis (breast infection) 7. Other | 1, 13 |
| Preterm Birth  (<37 weeks of gestation) | How long did this pregnancy last? | 1. Less than 8 weeks 2. 8-11 weeks 3. 12-19 weeks 4. 20-27 weeks 5. 28-31 weeks 6. 32-36 weeks 7. 37-39 weeks 8. 40-42 weeks (term) 9. 43+ weeks | 1, 13 |
| Pregnancy outcome | What was the outcome of your pregnancy that ended in [date]? | 1. Single live birth 2. Twins 3. Triplets + 4. Miscarriage/Stillbirth 5. Induced abortion 6. Tubal or ectopic | 1.1, 1.2, 1.3, 1.4, 1.5, 13 |
| Birth weight (used to calculate LBW and macrosomia) | Birth Weight (For multiples, please consider all babies) | 1. <5lbs 2. 5-5.4lbs 3. 5.5-6.9lbs 4. 7-8.4lbs 5. 8.5-9.9lbs 6. 10+lbs | Mod 1.1, 1.2 |
| Birth weight (used to calculate LBW and macrosomia) | **1.3** Birth Weight (For multiples, select a weight for each baby.) Your best guess is fine.  **1.4** For this pregnancy please enter your best guess for your baby's birth weight.  **1.5** Please enter your best guess for your baby's birth weight.  **13** Please enter your best guess for your baby's birth weight. | 1. Less than 3lbs 2. 3lbs-3lbs 8oz 3. 3lbs 9oz-3lbs 15oz 4. 4lbs-4lbs 8oz 5. 4lbs 9oz-4lbs 15oz 6. 5lbs-5lbs 8oz 7. 5lbs 9oz-5lbs 15oz 8. 6lb - 6lb 8oz 9. 6lb 9oz - 6lb 15oz 10. 7lb - 7lb 8oz 11. 7lb 9oz - 7lb 15oz 12. 8lb - 8lb 8oz 13. 8lb 9oz - 8lb 15oz 14. 9lb - 9lb 9oz 15. 9lb 9oz - 9lb 15oz 16. 10 or more lbs | 1.3, 1.4, 1.5, 13 |
| Birth weight (added ounces and pounds to calculate LBW and macrosomia) | Pounds | 1. 1 pound 2. 2 pounds 3. 3 pounds 4. 4 pounds 5. 5 pounds 6. 6 pounds 7. 7 pounds 8. 8 pounds 9. 9 pounds 10. 10 pounds 11. 11 pounds 12. 12 pounds 13. 13+ pounds | 1.5, 13 |
| Birth weight (Added ounces and pounds to calculate LBW and macrosomia) | Ounces | 1. 0 ounces 2. 1 ounce 3. 2 ounces 4. 3 ounces 5. 4 ounces 6. 5 ounces 7. 6 ounces 8. 7 ounces 9. 8 ounces 10. 9 ounces 11. 10 ounces 12. 11 ounces 13. 12 ounces 14. 13 ounces 15. 14 ounces 16. 15 ounces | 1.5, 13 |

^a^ Participants who left the PTSD question blank on all surveys completed were classified as not having PTSD. If a participant did not receive any survey version that included the question, they were assigned NA

Table S2. Risk ratios of adverse pregnancy outcomes by PTSD symptoms^a^ across sexual orientation groups adjusted for age and year of pregnancy.

|  | Completely heterosexual^b^(reference)  (n= 10,783) | Sexual minority^c^  (n=7,016) | Heterosexual with same-sex experience^d^ (n=2,864) | Mostly heterosexual (n=3,268) | Bisexual  (n=699) | Lesbian  (n=185) |
| --- | --- | --- | --- | --- | --- | --- |
| No PTSD Symptoms  Risk ratio (95% confidence interval) | | | | | | |
| Gestational diabetes^e^ (n=5,535) | 1.00 (ref) | 0.86 (0.64, 1.14) | 0.91 (0.62, 1.33) | 0.82 (0.55, 1.24) | 0.67 (0.25, 1.83) | 0.92 (0.32, 2.69) |
| Gestational hypertension^f^ (n=5,401) | 1.00 (ref) | 1.36 (1.06, 1.75) | 1.35 (0.97, 1.87) | 1.26 (0.89, 1.80) | 1.56 (0.67, 3.60) | 2.81 (1.24, 6.34) |
| Preeclampsia^g^ (n=5,636) | 1.00 (ref) | 0.96 (0.70, 1.33) | 0.82 (0.52, 1.29) | 1.15 (0.75, 1.77) | 0.85 (0.26, 2.75) | 0.92 (0.13, 6.32) |
| Preterm birth (n=5,725) | 1.00 (ref) | 0.89 (0.69, 1.16) | 0.84 (0.59, 1.19) | 0.88 (0.60, 1.27) | 1.17 (0.48, 2.82) | 1.58 (0.52, 4.79) |
| Low birthweight (n=5,705) | 1.00 (ref) | 0.99 (0.71, 1.38) | 1.04 (0.67, 1.60) | 0.80 (0.49, 1.32) | 2.02 (0.89, 4.59) | 1.04 (0.15, 7.08) |
| Macrosomia (n=5,705) | 1.00 (ref) | 1.02 (0.90, 1.17) | 0.92 (0.77, 1.11) | 1.16 (0.97, 1.39) | 0.91 (0.56, 1.49) | 0.91 (0.44, 1.89) |
| PTSD Symptoms  Risk ratio (95% confidence interval) | | | | | | |
| Gestational diabetes^e^ (n= 8,371) | 1.00 (ref) | 0.96 (0.80, 1.15) | 0.85 (0.65, 1.12) | 0.97 (0.77, 1.22) | 1.24 (0.86, 1.81) | 1.04 (0.48, 2.25) |
| Gestational hypertension^f^ (n= 8,058) | 1.00 (ref) | 1.11 (0.92, 1.33) | 1.21 (0.94, 1.54) | 1.04 (0.83, 1.32) | 0.99 (0.64, 1.53) | 1.44 (0.75, 2.75) |
| Preeclampsia (n= 8,501) | 1.00 (ref) | 1.19 (0.99, 1.42) | 1.51 (1.21, 1.89) | 1.00 (0.79, 1.28) | 0.85 (0.52, 1.37) | 1.34 (0.65, 2.75) |
| Preterm birth (n= 8,683) | 1.00 (ref) | 1.03 (0.88, 1.21) | 1.23 (1.00, 1.51) | 0.89 (0.72, 1.11) | 1.02 (0.71, 1.47) | 0.83 (0.41, 1.70) |
| Low birthweight (n= 8,639) | 1.00 (ref) | 1.27 (1.02, 1.57) | 1.29 (0.97, 1.71) | 1.24 (0.94, 1.62) | 1.26 (0.77, 2.06) | 1.60 (0.73, 3.49) |
| Macrosomia^g^ (n= 8,639) | 1.00 (ref) | 0.97 (0.88, 1.06) | 0.88 (0.77, 1.01) | 1.00 (0.88, 1.13) | 0.94 (0.74, 1.19) | 1.52 (1.10, 2.09) |
| ^a^PTSD symptoms prior to pregnancy were assessed using modified PTSD Checklist for DSM-5, in reference to the participant’s self-identified worst traumatic event  ^b^Completely heterosexual are participants who never had same-sex attractions, partners, or prior sexual minority identity  ^c^sexual minority includes the following subgroups: heterosexual with same-sex experience, mostly heterosexual, bisexual, and lesbian/gay  ^d^Heterosexual with same-sex experience are participants who reported “completely heterosexual” and either a prior sexual minority identity; having sexual contact with people who were the same-sex or another gender (e.g. gender fluid, non-binary); or being attracted to people of the same-sex or another gender (e.g. gender fluid, non-binary)  ^e^Restricted to pregnancies at ≥20 weeks’ gestation, with diagnosis year no history of chronic or gestational diabetes  ^f^Restricted to pregnancies at ≥20 weeks’ gestation, with diagnosis year and no history of chronic hypertension or gestational hypertension  ^g^Restricted to pregnancies at ≥20 weeks’ gestation, with diagnosis year and no history of preeclampsia | | | | | | |

Table S3. Risk ratios of adverse pregnancy outcomes by PTSD symptoms^a^ (<3 vs. ≥3 Symptoms) across sexual orientation groups

|  | Completely heterosexual^b^  (reference)  (n=11,762) | Sexual minority^c^  (n=7,471) | Heterosexual with same-sex experience^d^ (n=3,107) | Mostly heterosexual (n=3,438) | Bisexual  (n=729) | Lesbian  (n=197) |
| --- | --- | --- | --- | --- | --- | --- |
| < 3 PTSD Symptoms  Risk ratio (95% confidence interval) | | | | | | |
| Gestational diabetes^e^ (n=8,088) | 1.00 (ref) | 0.93 (0.74, 1.16) | 0.91 (0.67, 1.23) | 0.99 (0.73, 1.35) | 0.63 (0.28, 1.43) | 0.74 (0.25, 2.17) |
| Gestational hypertension^f^ (n=7,912) | 1.00 (ref) | 1.23 (0.99, 1.52) | 1.28 (0.98, 1.68) | 1.08 (0.80, 1.45) | 1.22 (0.59, 2.53) | 3.13 (1.59, 6.14) |
| Preeclampsia^g^ (n=8,234) | 1.00 (ref) | 1.24 (0.98, 1.56) | 1.30 (0.97, 1.74) | 1.17 (0.85, 1.61) | 1.49 (0.74, 3.00) | 0.63 (0.09, 4.32) |
| Preterm birth (n=8,374) | 1.00 (ref) | 0.95 (0.78, 1.15) | 1.01 (0.78, 1.31) | 0.84 (0.64, 1.12) | 1.07 (0.53, 2.16) | 1.50 (0.61, 3.67) |
| Low birthweight (n=8,336) | 1.00 (ref) | 1.06 (0.82, 1.37) | 1.16 (0.83, 1.62) | 0.82 (0.55, 1.21) | 1.91 (0.94, 3.88) | 1.44 (0.42, 4.96) |
| Macrosomia (n=8,336) | 1.00 (ref) | 0.95 (0.85, 1.06) | 0.89 (0.77, 1.04) | 1.02 (0.88, 1.18) | 0.82 (0.54, 1.24) | 1.15 (0.66, 2.00) |
| 3 or more PTSD Symptoms  Risk ratio (95% confidence interval) | | | | | | |
| Gestational diabetes^e^ (n=6,428) | 1.00 (ref) | 0.97 (0.79, 1.20) | 0.86 (0.64, 1.17) | 0.95 (0.73, 1.24) | 1.31 (0.88, 1.95) | 1.14 (0.52, 2.49) |
| Gestational hypertension^f^ (n=6,142) | 1.00 (ref) | 1.09 (0.89, 1.33) | 1.16 (0.88, 1.53) | 1.05 (0.81, 1.35) | 0.99 (0.63, 1.54) | 1.28 (0.64, 2.57) |
| Preeclampsia^g^ (n=6,507) | 1.00 (ref) | 1.08 (0.87, 1.32) | 1.34 (1.03, 1.73) | 0.95 (0.73, 1.25) | 0.74 (0.44, 1.25) | 1.36 (0.66, 2.81) |
| Preterm birth (n=6,651) | 1.00 (ref) | 0.98 (0.82, 1.18) | 1.18 (0.93, 1.49) | 0.87 (0.68, 1.11) | 0.93 (0.64, 1.35) | 0.76 (0.33, 1.71) |
| Low birthweight (n=6,614) | 1.00 (ref) | 1.18 (0.93, 1.50) | 1.14 (0.82, 1.57) | 1.23 (0.92, 1.64) | 1.08 (0.64, 1.82) | 1.37 (0.56, 3.35) |
| Macrosomia (n=6,614) | 1.00 (ref) | 0.98 (0.88, 1.09) | 0.94 (0.80, 1.09) | 0.99 (0.86, 1.13) | 0.90 (0.70, 1.15) | 1.56 (1.12, 2.17) |
| ^a^PTSD symptoms prior to pregnancy were assessed using modified PTSD Checklist for DSM-5, in reference to the participant’s self-identified worst traumatic event  ^b^Completely heterosexual are participants who never had same-sex attractions, partners, or prior sexual minority identity  ^c^sexual minority includes the following subgroups: heterosexual with same-sex experience, mostly heterosexual, bisexual, and lesbian/gay  ^d^Heterosexual with same-sex experience are participants who reported “completely heterosexual” and either a prior sexual minority identity; having sexual contact with people who were the same-sex or another gender (e.g. gender fluid, non-binary); or being attracted to people of the same-sex or another gender (e.g. gender fluid, non-binary)  ^e^Restricted to pregnancies at ≥20 weeks’ gestation, with no history of chronic or gestational diabetes or year Isof diagnosis  ^f^Restricted to pregnancies at ≥20 weeks’ gestation, with no history of chronic hypertension or gestational hypertension or year of diagnosis  ^g^Restricted to pregnancies at ≥20 weeks’ gestation, with no history of preeclampsia or year of diagnosis | | | | | | |

Table S4. Risk ratios of adverse pregnancy outcomes by PTSD diagnosis^a^ across sexual orientation groups.

|  | Completely heterosexual^b^  (reference)  (n=15,642) | Sexual minority^c^  (n= 10,759) | Heterosexual with same-sex experience^d^ (n= 4,705) | Mostly heterosexual (n=4,773) | Bisexual  (n=1,021) | Lesbian  (n=260) |
| --- | --- | --- | --- | --- | --- | --- |
| No PTSD Diagnosis  Risk ratio (95% confidence interval) | | | | | | |
| Gestational diabetes^e^ (n=18,214) | 1.00 (ref) | 1.00 (0.87, 1.15) | 0.92 (0.76, 1.11) | 0.99 (0.83, 1.20) | 1.39 (1.02, 1.90) | 1.15 (0.64, 2.08) |
| Gestational hypertension^f^ (n=17,669) | 1.00 (ref) | 1.17 (1.03, 1.33) | 1.20 (1.01, 1.43) | 1.09 (0.91, 1.29) | 1.30 (0.95, 1.78) | 1.53 (0.93, 2.52) |
| Preeclampsia^g^ (n=18,477) | 1.00 (ref) | 1.22 (1.06, 1.40) | 1.41 (1.19, 1.67) | 1.10 (0.91, 1.32) | 0.99 (0.67, 1.47) | 0.94 (0.45, 1.97) |
| Preterm birth (n=18,796) | 1.00 (ref) | 0.98 (0.87, 1.10) | 1.03 (0.89, 1.19) | 0.89 (0.76, 1.05) | 1.12 (0.85, 1.48) | 1.10 (0.65, 1.87) |
| Low birthweight (n=18,619) | 1.00 (ref) | 1.14 (0.98, 1.32) | 1.13 (0.93, 1.38) | 1.05 (0.86, 1.29) | 1.44 (1.00, 2.06) | 1.60 (0.87, 2.95) |
| Macrosomia (n=18,619) | 1.00 (ref) | 0.95 (0.89, 1.01) | 0.92 (0.84, 1.01) | 0.96 (0.87 1.05) | 0.90 (0.74, 1.09) | 1.38 (1.07, 1.79) |
| PTSD Diagnosis  Risk ratio (95% confidence interval) | | | | | | |
| Gestational diabetes^e^ (n=68) | 1.00 (ref) | 1.54 (0.20, 11.80) | 3.75 (0.44, 32.26) | 1.79 (0.21, 15.13) | -- | -- |
| Gestational hypertension^f^ (n=54) | 1.00 (ref) | 1.46 (0.19, 10.90) | -- | 1.89 (0.25, 14.56) | 1.14 (0.9, 15.08) | -- |
| Preeclampsia^g^ (n=68) | 1.00 (ref) | 1.27 (0.27, 6.06) | 0.92 (0.11, 7.95) | 1.57 (0.30, 8.35) | 0.92 (0.09, 8.86) | -- |
| Preterm birth (n=68) | 1.00 (ref) | 0.46 (0.10, 2.03) | 0.37 (0.05, 2.78) | 0.74 (0.16, 3.48) | -- | -- |
| Low birthweight (n=68) | 1.00 (ref) | 1.97 (0.42, 9.11) | 1.11 (0.13, 9.56) | 2.86 (0.58, 14.08) | 0.83 (0.09, 7.99) | -- |
| Macrosomia (n=68) | 1.00 (ref) | 0.75 (0.22, 2.54) | 1.25 (0.30, 5.17) | 0.95 (0.26, 3.52) | 0.16 (0.02, 1.28) | -- |
| ^a^PTSD diagnosis prior to pregnancy includes clinician-diagnosed PTSD within the past three years or any prior diagnosis more than three years ago  ^b^Completely heterosexual are participants who never had same-sex attractions, partners, or prior sexual minority identity  ^c^sexual minority includes the following subgroups: heterosexual with same-sex experience, mostly heterosexual, bisexual, and lesbian/gay  ^d^Heterosexual with same-sex experience are participants who reported “completely heterosexual” and either a prior sexual minority identity; having sexual contact with people who were the same-sex or another gender (e.g. gender fluid, non-binary); or being attracted to people of the same-sex or another gender (e.g. gender fluid, non-binary)  ^e^Restricted to pregnancies at ≥20 weeks’ gestation, with diagnosis year and no history of chronic or gestational diabetes or year of diagnosis  ^f^Restricted to pregnancies at ≥20 weeks’ gestation, with diagnosis year and no history of chronic hypertension or gestational hypertension or year of diagnosis  ^g^Restricted to pregnancies at ≥20 weeks’ gestation, with diagnosis year and no history of preeclampsia or year of diagnosis | | | | | | |
